# Going beyond routine data collection of clinical trials funded by the health technology assessment programme

**DOI:** 10.1186/1745-6215-16-S2-P42

**Published:** 2015-11-16

**Authors:** James Raftery, Amanda Young, Helen Buxton

**Affiliations:** 1 University of Southampton and University Hospital Southampton, Southampton, UK; 2 National Institute for Health Research (NIHR), Evaluation, Trials and Studies Coordinating Centre (NETSCC), University of Southampton, Southampton, UK

## Background

In 2015, a HTA funded project developed and piloted a database to capture ‘metadata’ on published HTA clinical trials in six priority areas: origins of the topic, the conduct and performance of trials, the statistical and economic analyses, and the cost of trials. The report recommendations were to expand, amend and update the database in terms of the number of trials and amount of data extracted.

## Aims and objectives

To amend and update the database based on the published recommendations, explore trends over time and develop an NIHR portfolio database of active [pre-contracting to submission of final report] clinical trials.

## Method

We reviewed the HTA portfolio for RCTs that had published in the HTA Journal Library since February 2011 to December 2014. The 65 questions which were recommended to be readily extracted, were reviewed and updated. RCTs published to December 2012 were used to pilot any alterations.

## Results

The key findings will include all HTA funded clinical trials published to end December 2014. Based on previous work, data will be presented under the six priority areas such as the design of the trial, recruitment targets met/unmet and differences in results between new and old interventions.

## Conclusions

Expanding and updating the metadata database will provide important feedback about the quality of performance and reporting of HTA funded clinical trials. The continuation of the database will provide robust evidence of the lifecycle of funded trials from source of commissioning through to publications, including factors associated to what was originally planned.

